# Nucleation and growth dynamics of graphene grown by radio frequency plasma-enhanced chemical vapor deposition

**DOI:** 10.1038/s41598-021-85537-3

**Published:** 2021-03-16

**Authors:** Na Li, Zhen Zhen, Rujing Zhang, Zhenhua Xu, Zhen Zheng, Limin He

**Affiliations:** 1 Key Laboratory of Advanced Corrosion and Protection for Aviation Materials, Beijing Institute of Aeronautical Materials, Aero Engine Corporation of China, Beijing, 10095 China; 2 Beijing Institute of Graphene Technology, Beijing, China

**Keywords:** Nanoscale materials, Chemical engineering

## Abstract

We investigated the nucleation and grain growth of graphene grown on Cu through radio frequency plasma-enhanced chemical vapor deposition (RF-PECVD) at different temperatures. A reasonable shielding method for the placement of copper was employed to achieve graphene by RF-PECVD. The nucleation and growth of graphene grains during PECVD were strongly temperature dependent. A high growth temperature facilitated the growth of polycrystalline graphene grains with a large size (~ 2 μm), whereas low temperature induced the formation of nanocrystalline grains. At a moderate temperature (790 to 850 °C), both nanocrystalline and micron-scale polycrystalline graphene grew simultaneously on Cu within 60 s with 50 W RF plasma power. As the growth time increased, the large graphene grains preferentially nucleated and grew rapidly, followed by the nucleation and growth of nanograins. There was competition between the growth of the two grain sizes. In addition, a model of graphene nucleation and grain growth during PECVD at different temperatures was established.

## Introduction

Among the nanomaterials discovered to date, graphene is the thinnest, with the highest strength and electrical and thermal conductivities. These unique properties make graphene a very useful novel material^1–4^. In-depth research has been conducted on the basic preparation, performance optimization, and R&D applications of graphene^5–10^. The synthesis of high-quality graphene with excellent performance is a prerequisite for many applications and therefore a core technology for research. A variety technologies for graphene preparation have been developed to meet the needs of practical applications and in-depth research, including mechanical exfoliation^1^, epitaxial growth^11^, oxidation-reduction^12, 13^, chemical vapor deposition (CVD)^14–17^, and so on. Due to its excellent controllability and low cost, CVD is one of the most effective methods for preparing high-quality graphene. CVD can be classified into thermal CVD and plasma-enhanced CVD (PECVD) depending on the mode of energy supplied for grain growth. Traditional thermal CVD involves heating a substrate to the cracking temperature of the carbon (C) source (typically above 1000 °C) and using dissolution-precipitation^18, 19^ or adsorption^14^ of C on a metal substrate to achieve graphene. The high-temperature growth environment required for this method limits substrate preparation and deteriorates the graphene quality during the cooling process^20, 21^. In addition, the long growth cycle of thermal CVD is not conducive to industrial production.

PECVD offers the advantage of a lower preparation temperature than CVD. Plasma contains a large number of high-energy electrons. Collisions between electrons and gas-phase molecules promote the breakage and recombination of chemical bonds of reactant gas molecules to generate chemical species with high activities. High-energy electrons provide the activation energy required for the CVD process. The mode of energy supply for the reaction system in PECVD is different from that in CVD and enables the entire reaction to proceed at lower temperatures. Therefore, PECVD shows considerable potential for low-temperature graphene preparation. Researchers have achieved the low-temperature growth of graphene thin films by PECVD on transition metal substrates such as nickel^22–24^, copper (Cu)^25–27^, and cobalt^28^, dielectric substrates^29–31^, and other 2-dimensional materials^32^.

More complex reactions occur during the PECVD process than those during thermal CVD. The plasma can promote the decomposition of hydrocarbon to generate a large number of free radicals and active species, which have extremely high densities and activities and rapidly react on the substrate surface, resulting in the fast nucleation of graphene and a slow growth rate. The final graphene film may have problems such as a small grain size, excessive edges, or the formation of amorphous carbon, which significantly deteriorate the electrical performance of graphene. The electron mobility of graphene prepared by PECVD is much lower than that of graphene films prepared by thermal CVD^33^, which considerably limits applications in electronic devices. Previous studies have shown that factors such as the plasma energy^34, 35^, C/H ratio^36, 37^, and temperature^22, 38, 39^ play a significant role in graphene nucleation and grain growth during the PECVD process; among them, the effects of the growth temperature on nucleation and growth were reported to be crucial. Liu et al. revealed that changing the growth temperature induced a nanocrystalline-to-polycrystalline transition of graphene and that nucleation but no graphene grain growth occurred at a specific temperature and vice versa^38^. Recently, Yen et al. studied the kinetics of nucleation and growth during PECVD and found that the graphene nucleation density and grain size changed with the temperature^25^.

However, in previous studies, there was only one type of graphene nucleation and growth at a specified growth temperature, either nanocrystalline or micron-sized polycrystalline. In this study, a surprising finding that was both nanocrystalline and micron-sized polycrystalline graphene could grow simultaneously during RF-PECVD under certain temperature conditions. Consequently, we investigated the effect of the growth temperature on the nucleation and growth of these two kinds of graphene grains and verified the criticality of temperature for the appearance of nanocrystalline and micron-size polycrystalline graphene. The growth processes of the two graphene grains were observed during different growth times, and a model of graphene nucleation and grain growth at different temperatures during PECVD was established.

## Experimental

### Pretreatment of the Cu substrate

**Figure 1:**
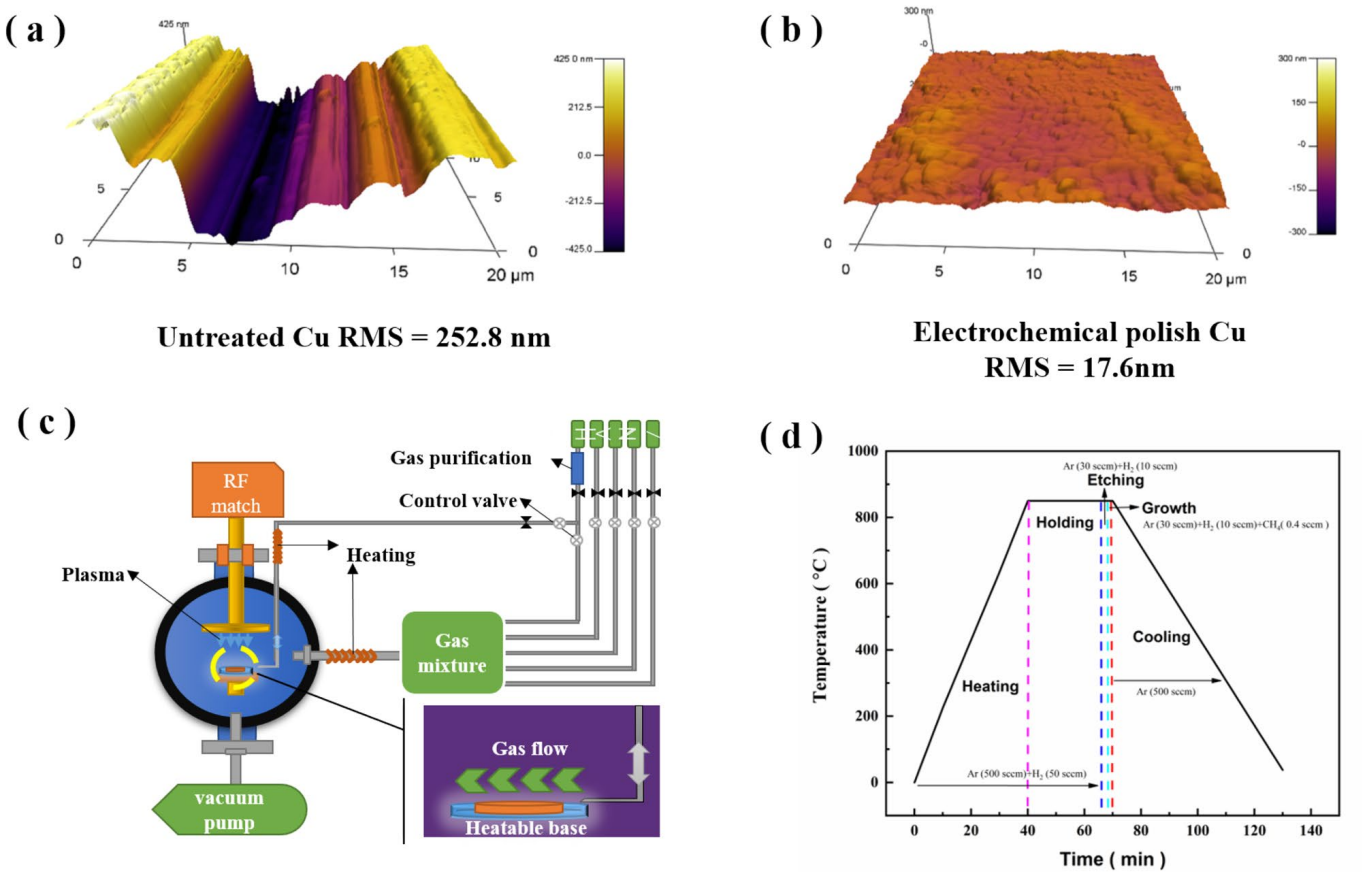
3D AFM images of the (**a**) untreated Cu surface and (**b**) Cu surface after electrochemical polishing pretreatment. The RMS of the untreated and pretreated Cu surface was 252.8 nm and 17.6 nm, respectively. (**c**) Schematic diagram of the ICP-CVD apparatus with an RF radio frequency generator. (**d**) The graphene growth processes, including heating, holding, etching, growth, and cooling.

Alfa Cu foil (Alfa Aesar no. 13382, 25 µm, 99.8%) was used as the substrate for graphene growth. The Cu foil was cut to 2 cm × 2 cm and ultrasonically cleaned sequentially using hydrochloric acid (5 wt%), acetone, and isopropanol for 5 min to remove the oxide layer and organic impurities on the surface. The Cu foil was then dried with nitrogen. There have been extensive reports that the Cu foil surface roughness significantly affects the nucleation and growth of CVD-grown graphene. Cu foil with a low surface roughness and high flatness promotes the growth of large grains. Therefore, the Cu foil was pretreated by electrochemical polishing to obtain a flat surface on which to grow graphene by PECVD. The electrochemical polishing was performed at a bias of 1.9 V in a phosphoric acid electrolyte (H_3_PO_4_:H_2_O = 2:1) for 5 min. The Cu foil was then rinsed with deionized water for 5 min and blown dry with nitrogen. Atomic force microscopy (AFM) was used to characterize the surface morphology and roughness of the Cu substrate after different pretreatments. Figure 1a shows the 3-dimensional surface morphology of the untreated Cu foil, which was characterized by large undulations from pressing patterns produced during the manufacturing process. The surface root-mean-square (RMS) height was approximately 252.8 nm. Electrochemical polishing largely eliminated the pressing patterns on the Cu surface and reduced the surface undulations. The surface RMS height was 17.6 nm, as shown in Fig. 1b. Thus, the pretreatment of electrochemical polishing could effectively reduce the surface roughness of Cu, which might facilitate the growth of graphene during PECVD.

### PECVD growth and transfer of graphene

Graphene was prepared in a radio frequency PECVD (RF-PECVD) apparatus, which was mainly composed of a gas delivery system, a plasma device, a heating system, and a vacuum system. A schematic of the apparatus is shown in Fig. 1c. The pretreated Cu foil was placed in a quartz supporter that was then placed in a quartz tube. The quartz tube was sealed, the vacuum system was turned on, and the system pressure was pumped down to below 0 Pa. The vacuum pump was turned off, and 1000 sccm argon (Ar) gas was introduced until the system pressure reached ambient pressure to exhaust the residual air in the tube. The Ar gas supply was turned off, and the vacuum pump was turned on again to evacuate the system to 0 Pa. The system was heated to the target temperature (670–850 °c) at 230 Pa in a mixture of 500 sccm Ar and 50 sccm H_2_. After the target temperature was reached, the Ar and H_2_ gas flow rates were adjusted to 30 sccm and 10 sccm, respectively, and the system pressure was maintained at 43 Pa. After the gas flow stabilized, the plasma (50 W) was turned on, and etching was performed for 1 min. Immediately afterwards, 8 sccm CH_4_ (methane)/Ar (5% by volume) was introduced for graphene growth. Growth times of 10 s, 20 s, 30 s, 40 s, 60 s, and 90 s were used. When growth was completed, the CH_4_/Ar mixed gas, H_2_, plasma, and heating system were turned off. The quartz shield and Cu foil were quickly removed from the reactor. The temperature was reduced to room temperature in 500 sccm Ar. The vacuum system was turned off, and 500 sccm Ar was continuously passed through the system until the system pressure reached ambient pressure. Finally, the Ar gas supply was terminated, and the sample was removed from the apparatus. Figure 1d shows the growth process of graphene by PECVD at 850 °C for 60 s.

The structure and properties of the prepared graphene were subsequently characterized. A polymethyl methacrylate (PMMA)-protected transfer method was used to transfer the graphene to silicon dioxide/silicone (SiO_2_/Si). First, PMMA was uniformly spin-coated on the surface of the graphene grown on Cu foil as a protective layer, followed by curing in a vacuum-drying oven at 140 °C for 30 min. The sample was then placed in an iron(III) chloride (FeCl_3_) solution (5% by mass). After the Cu foil was completely etched, the graphene/PMMA film was transferred to deionized water using a glass sheet, followed by rinsing 3 times to remove residual chemical impurity ions from the sample surface. SiO_2_/Si was used to pick up the sample. The surface PMMA was removed using acetone and isopropanol to produce graphene on SiO_2_/Si.

### Characterization of graphene

Tapping-mode AFM (Oxford Cypher VRS AFM system) was performed to characterize the surface morphology of the Cu foil before and after the electrochemical polishing pretreatment and the morphology and thickness of the graphene on SiO_2_/Si. The nucleation density, grain size, and morphology of the prepared graphene were characterized using optical microscopy (Axio scope A1) and scanning electron microscopy (SEM). The SEM images were acquired by using an FEI Nova Nano450-SEM instrument at 10 kV. The layer numbers and defects of the graphene were identified by Raman spectroscopy (Horiba Evolution) with a 532-nm laser.

## Results and discussion

### The placement of the copper foil for graphene growth

**Figure 2:**
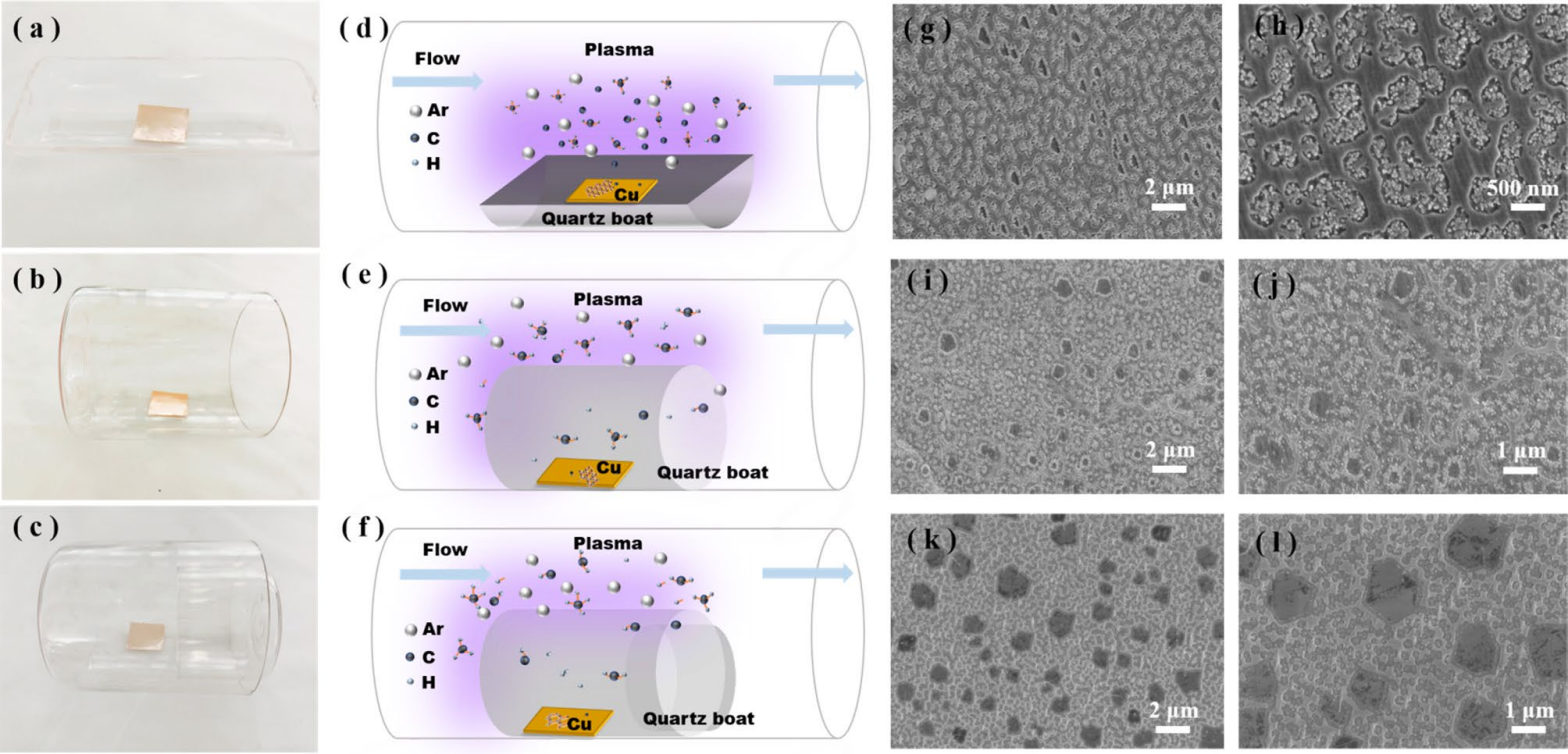
(**a**–**c**) Images of different quartz boat placements for graphene growth on Cu. (**d**–**f**) Schematic diagram of graphene growth with different placement methods. (**g**–**h**), (**i**–**j**), and (**k**–**l**) SEM photographs of graphene growing with placement modes (**a**–**c**), respectively. Graphene was grown on copper substrate at 850 °C for 60 s with Ar/H_2_/CH_4_ = 30:10:0.4. The plasma power was 50 W.

Plasma can effectively promote the decomposition of hydrocarbon precursor, and a mass of plasma-generated ions and radicals, such as Ar ions, cause drastic bombardment^26^. In addition, high densities of H atoms and other free radicals cause excessive etching of graphene^34, 40^. Therefore, we employed three different placement methods to grow graphene. The schematics of the three different substrate placement modes and the corresponding PECVD-grown graphene are shown in Fig. 2a–f. The other growth conditions remained unchanged. In the first placement mode shown in Fig. 2a,d, the entire surface of the Cu foil was exposed to the plasma. The plasma and Cu foil jointly promoted the decomposition of hydrocarbon precursor, generating a high density of free atoms and active species on the Cu surface, which resulted in excess ion bombardment. Figure 2g,h show the surface defects caused by ion bombardment, which significantly deteriorated the structural integrity of the grown graphene. In the second placement mode, a semiclosed quartz shield with an 80-mm diameter was used, as shown in Fig. 2b,e. The purpose of this placement method was to weaken high-energy Ar ion bombardment and reduce the excessive etching effect of plasma with a high density of active species. Figure 2i,j show that the graphene prepared using the second placement method had considerably fewer defects from ion bombardment than that prepared with the first method. Incomplete hexagon-shaped 1-µm graphene grains were produced. Unfortunately, there still were distinct traces of ion bombardment and incomplete graphene grains. Thus, we designed a third substrate placement method that involved a semiclosed quartz shield with a 60-mm diameter to block the open end of the quartz shield shown in Fig. 2b, as presented in Fig. 2e,f. One of the main effects of this mode of loading was a change in the transport mode of reactant gas to the surface of the catalytic substrate and a reduction in the concentration of reactants on the substrate surface. The finite element analysis and simulation results show that under the condition of low-pressure CVD (LPCVD), the gas velocity in the confined space was very low and the diffusion flow was dominant, so the transport of reactants to the substrate surface was greatly reduced, which was conducive to the growth of graphene in the PECVD process. Figure 2k,l show that the graphene prepared using the third method had a regular hexagonal shape and the grain size was approximately 2 μm. The finite element analysis and simulation results show that under the condition of LPCVD, the gas velocity in the confined space was very low and the diffusion flow was dominant, so the transport of reactants to the substrate surface was greatly reduced, which was conducive to the growth of graphene in the PECVD process. Under our PECVD conditions, the third placement method for the Cu foil effectively reduced the damage to the graphene caused by ion bombardment and plasma etching.

### Features of the PECVD-grown graphene

**Figure 3:**
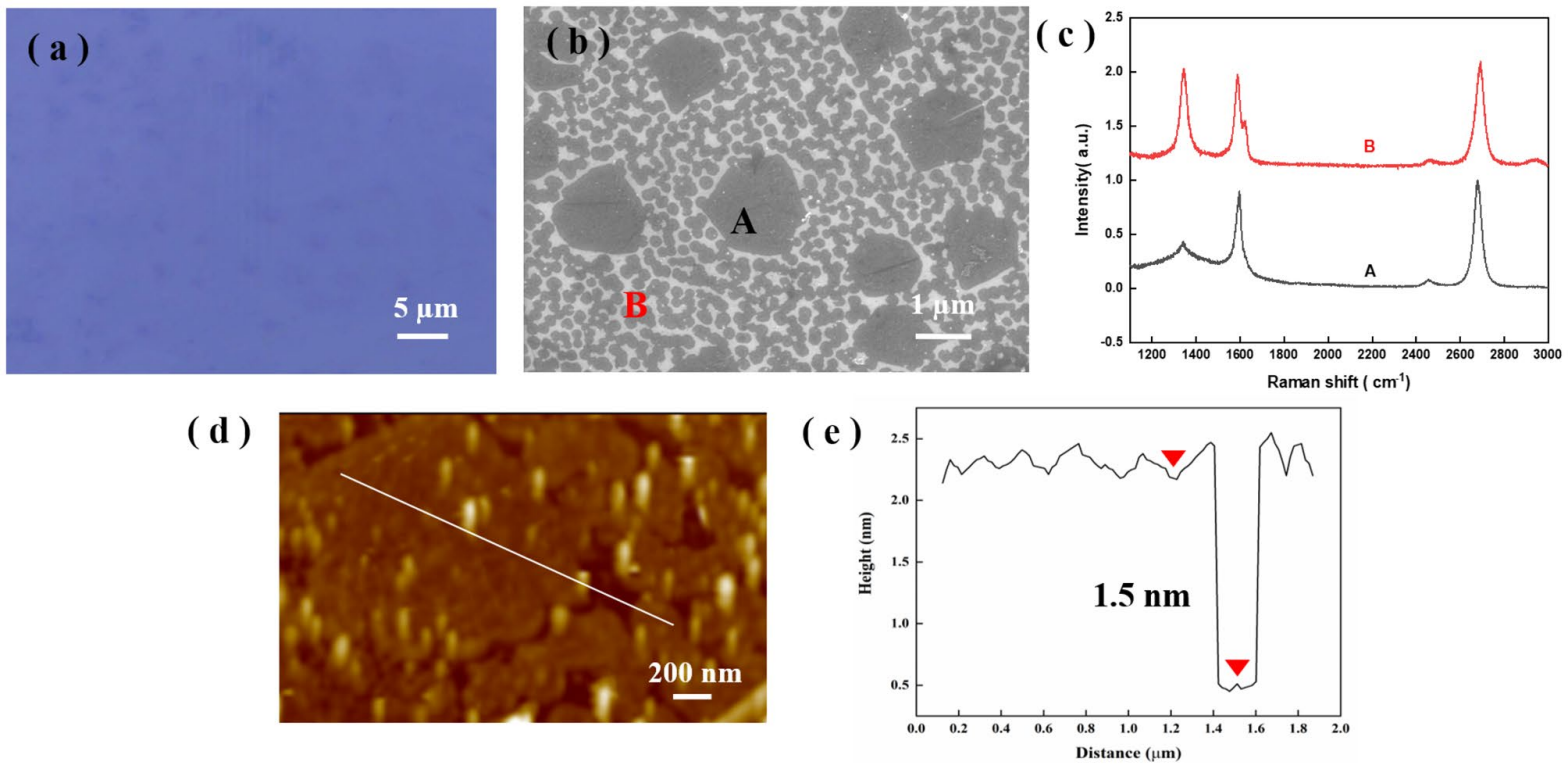
(**a**) Optical microscopy image and (**b**) SEM image of graphene transferred onto SiO_2_/Si. (**c**) Raman spectra at A and B in (**b**). (**d**) AFM image of graphene transferred onto SiO_2_/Si. (**e**) Height diagram of the white line in (**d**).

The characteristics of graphene grown using the third placement method were measured. Optical microscopy and SEM images of the graphene transferred onto SiO_2_/Si are shown in Fig. 3a,b, respectively. The as-grown graphene consists of two sizes, including a few polycrystalline graphene grains with an average size of 1.5 μm (area A) and a large number of 260-nm nanocrystalline grains (area B). Figure 3c shows the Raman spectroscopy results for areas A and B in Fig. 3b, which both correspond to graphene. The extremely weak D peak of the Raman spectrum in area A indicated that there were very few defects in this area. The I_2D_/I_G_ ratio of approximately 1.17 showed that there were few layers. The high-intensity D peak of the Raman spectrum in area B suggested there were a mass of edges and defects in area B, which were caused by the nanometer-sized grains of graphene. Figure 3d shows the AFM surface morphology of the graphene film. Micron-sized hexagonal graphene grains surrounded by round nanocrystalline graphene were observed. The thickness at the white line was approximately 1.5 nm, as shown in Fig. 3e, indicating that there were approximately 1 to 3 graphene layers. In summary, graphene with 2 grain sizes was obtained at 850 °C during our PECVD process after 60 s of growth. The as-grown graphene consisted of both micron-scale polycrystalline and nanocrystalline forms. We explored the dynamics of nucleation and grain growth of these two sizes of graphene grains during the PECVD process. The results provide guidance for the synthesis of high-quality, large-grain graphene by PECVD.

### Effects of temperature on nucleation and grain size

**Figure 4:**
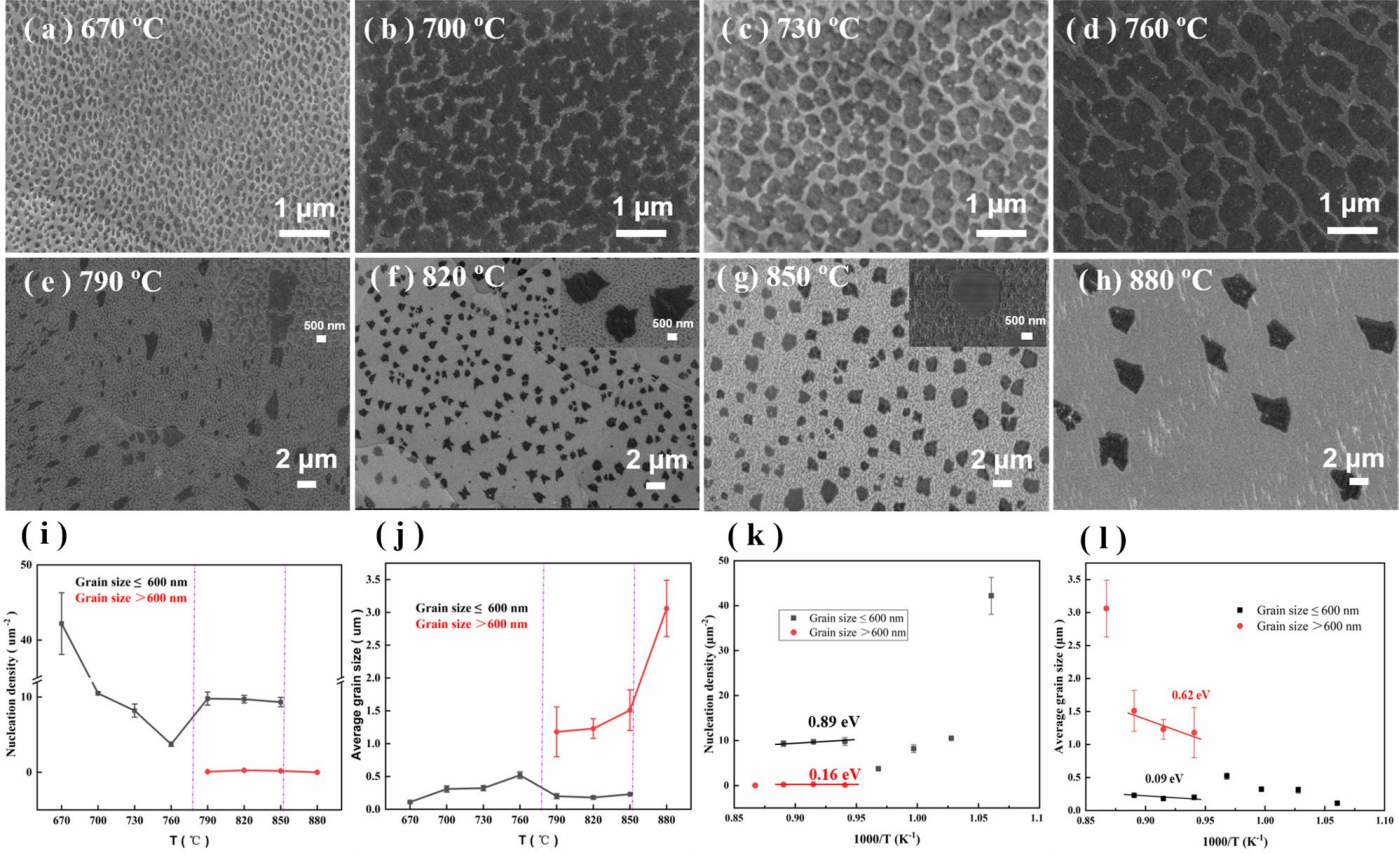
(**a**–**h**) SEM images of graphene grown on polished Cu at different temperatures from 670 °C to 880 °C for 60 s. The plasma power was 50 W. The (**i**) nucleation density and (**j**) grain size of graphene with the growth temperature. Arrhenius plots of the (**k**) nucleation density and (**l**) average grain size (the black line represents graphene grains < 600 nm and the red line represents grains > 600 nm).

The nucleation density and grain size of graphene grown on Cu by PECVD using the third placement method were studied at different temperatures. Figure 4a–h show SEM images of graphene grains synthesized at growth temperatures of 670 to 880 °C by PECVD. The remaining growth conditions were consistent for all samples: plasma power of 50 W, CH_4_/H_2_ gas ratio of 0.04, growth pressure of 44 Pa, and growth time of 60 s. In Fig. 4a–d, the SEM results show that only nanocrystalline graphene was obtained when the growth temperature was below 760 °C. We found that the average size of nanocrystalline graphene increased with the growth temperature. When the temperature increased from 670 to 760 °C, the nanocrystalline size increased from 100 to 520 nm. In contrast, the nucleation density decreased as the nanocrystalline size increased because of competition between nucleation and growth. In addition, we noted that the nanocrystalline graphene grown over this temperature range was uniform in size and distribution and mostly oval, suggesting the rapid nucleation of graphene and uniformity of nanocrystalline growth. When the growth temperature was increased to 790 to 850 °C, we simultaneously obtained graphene grains with two sizes, i.e., relatively large grains (600 nm to micron size) and small grains (less than 600 nm), as shown in Fig. 4e–g. Starting at 790 °C, a few grains with sizes of 1 to 2 μm and some grains with sizes of 600 nm to 1 μm appeared and coexisted with a large number of nanocrystalline grains ~ 2560 nm in size, as shown in Fig. 4e. As the temperature increased to 830 °C, the larger graphene grains (> 600 nm) enlarged to ~ 1.2 μm, and the resulting grain size and distribution of graphene grains were more uniform than those of the large grains (> 600 nm) at 790 °C, as shown in Fig. 4f. The coexisting nanocrystalline (< 600 nm) grains were not significantly different in size from those at 790 °C. At 850 °C and a 60-s growth time, the average grain size of the large graphene grains (> 600 nm) increased to approximately 1.5 μm, whereas the size of nanocrystalline graphene remained unchanged at approximately 260 nm, as shown in Fig. 4g. Notably, the large graphene grains (> 600 nm) and nanocrystalline graphene (< 600 nm) simultaneously nucleated and grew over the 790–850 °C temperature range. As the temperature increased, the large graphene grains (> 600 nm) expanded, whereas the nanocrystalline grains (< 600 nm) remained almost unchanged, suggesting that the formation of nanocrystalline graphene (< 600 nm) was not sensitive to temperature changes within the temperature range considered. Increasing the temperature further to 880 °C resulted in only large graphene grains (> 600 nm) and no coexisting nanocrystalline form (< 600 nm). The graphene grain size increased up to approximately 3 µm at 880 °C, as shown in Fig. 4h.

We investigated the dynamics of the nucleation and growth of graphene grains of two sizes; the nucleation density and grain sizes of the large graphene grains (> 600 nm) and nanocrystalline graphene grains (< 600 nm) are plotted as a function of the growth temperature in Fig. 4i,j. Only nanograins grew in the low-temperature region (670–760 °C) in our PECVD process. As the temperature increased, the nucleation density decreased and the average grain size increased. Large grains and nanocrystalline graphene coexisted in the moderate temperature region (790 to 850 °C). The nucleation density of the 2 grain sizes did not change significantly with temperature. However, the average grain size of the large grains increased with the temperature, whereas that of the nanograins remained almost unchanged. This result implies that within the moderate temperature range, the growth of nanocrystalline graphene was not sensitive to temperature changes. In the high-temperature region (≥ 880 °C), only large graphene grains nucleated and grew, and no nanograins formed. The graphene nucleation density was slightly lower than that in the moderate temperature region, and the average grain size was larger. The above analysis shows that the nucleation and growth of graphene grains during PECVD are strongly temperature dependent. A high temperature facilitates the growth of polycrystalline graphene grains with a large size, and low temperature induces a nanocrystalline form.

Generally, temperature has significant effects on the dehydrogenation of hydrocarbon precursors, the adsorption and diffusion of active carbon species on the copper substrate surface, and the surface roughness and cleanliness of copper. At high temperature, plasma and catalytic copper enhanced the supply of the active carbon species by promoting the dehydrogenation of hydrocarbon, which were effective to suppress the excess nucleation and to facilitate graphene growth^41^. Besides, During the high-temperature annealing process, the surface roughness of copper was improved, and the impurities on the copper surface and inside were reduced, which also reduced nucleation and promoted graphene grain growth. When the temperature was very low, there was less catalytic effect of the copper surface, and the dehydrogenation of hydrocarbon depended entirely on the plasma. Due to the high binding energy at low temperature, the nucleation of the active carbon species on the copper surface or adhesion to the edge of the graphene nucleus was determined by the mobility of active radicals on the copper surface, which was reduced at lower temperature. The nucleation rate was limited by capture of carbon adatoms by supercritical nucleus^42^. There is a critical state in the middle temperature region. The copper substrate surface did not catalyze as strongly as they did at high temperatures, but it was stronger than that of at low temperatures. Carbon atoms are more likely to coalesce and nucleate preferentially at high-energy sites such as the rough surface of the copper substrate, defect sites (grain boundaries, dislocations, steps, etc.) and impurities, as they did at high temperatures. Then, some of the subsequent carbon atoms coalesced at these nucleuses to make the grains grow because that subsequent carbon atoms were more likely to enrich at these places. As a result, there were enough carbon atoms to grow graphene for these nucleuses. On the other hand, the adsorption of carbon atoms also occurred elsewhere on the copper surface, but it was sporadic and random, so graphene growth at those sites were affected by the capture of carbon adatoms by supercritical nucleus, which was determined by the diffusion and migration of carbon atoms. The preference of nucleation was related to the energy difference at different sites on the surface of the copper substrate, which could be ignored at lower temperature due to the complete loss of copper catalysis.

### Arrhenius plots

The energetics of the growth were investigated by measuring the activation energies. Figure 4k,l show the Arrhenius plots for the nucleation density and grain size for the two kinds of graphene grains grown on Cu at a moderate temperature, respectively. The activation energy of the nucleation of large graphene grains (> 600 nm) was much lower than that of nanocrystalline graphene (< 600 nm), indicating that the nucleation of larger graphene grains was suppressed, possibly via competition for the capture of carbon adatoms by supercritical nucleus and the mobility of active carbon species on the copper substrate surface. Conversely, the activation energy for larger graphene grain sizes was higher than that for nanocrystalline graphene. This finding suggests that nucleation of large grains required lower reaction barriers, so it occurred easily at first, but further expansion of the grain size was more difficult. However, nanocrystals did the opposite. This result implies that the growth of large grains was a process based on thermodynamics, while the growth of small grains was more related to kinetic processes, which will be verified in a follow-up study.

### Growth dynamics

**Figure 5:**
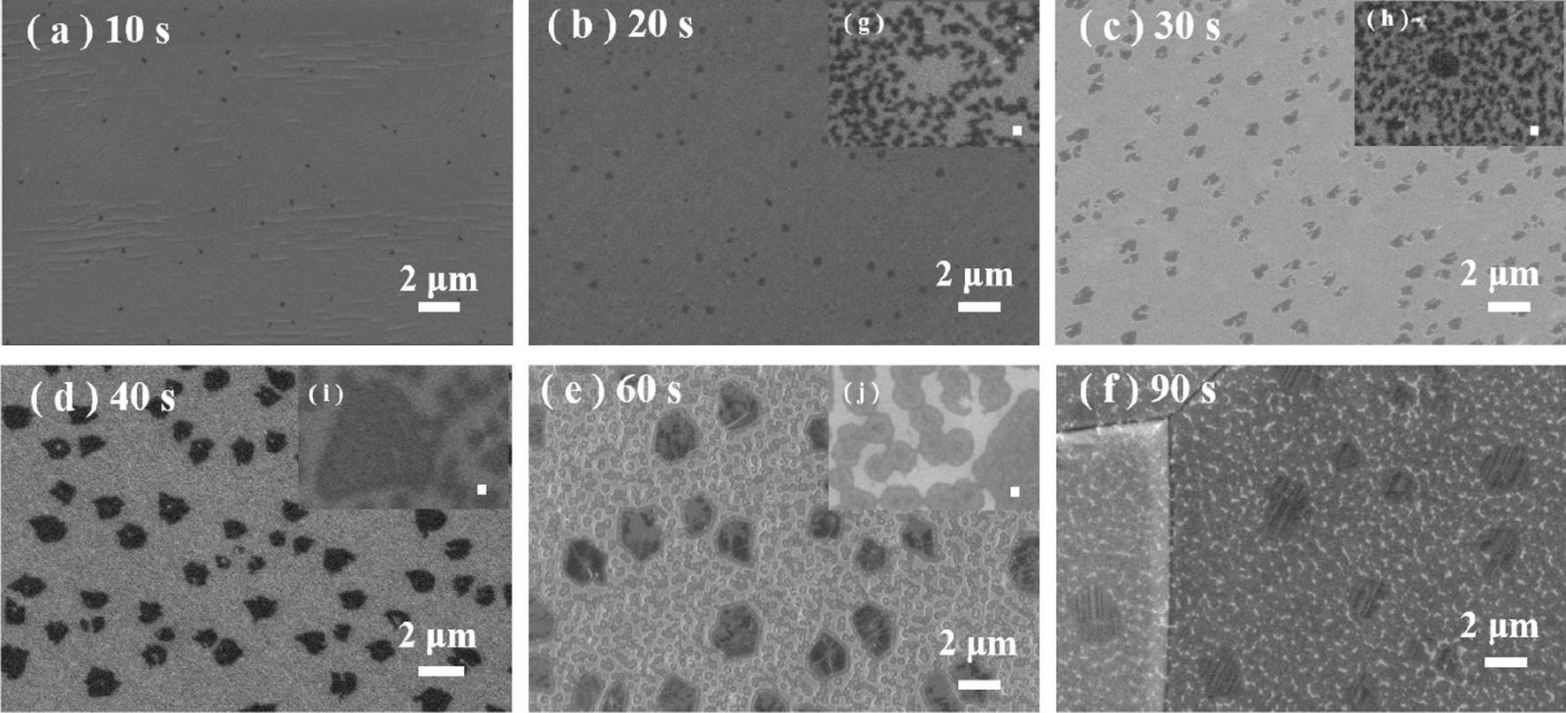
(a)–(f) SEM images of graphene growing on polished Cu at different growth times (10–90 s) at 850 °C with Ar:H_2_:CH_4_ = 30:10:0.4, 50 W. The scale bar is 2 µm. (**g**–**j**) SEM images of graphene transferred onto SiO_2_/Si for 20 s, 30 s, 40 s and 60 s, respectively. The scale bar is 100 nm.

PECVD was carried out at 850 °C for different growth times (10, 20, 30, 40, 60, and 90 s) to further investigate the growth process of large graphene grains and nanocrystalline graphene grains over the moderate temperature zone. All other conditions remained unchanged. Figure 5a–f show SEM images of graphene grains prepared by PECVD at different growth times. At 10 s, graphene nucleation ~ 150 nm in size appeared on the Cu surface, as shown in Fig 5a. As the growth time increased to 20 s, the graphene grain size increased to ~ 350 nm, as shown in Fig 5b, which was much larger than that of the coexisting nanograins (~ 260 nm) obtained under the same conditions for a 60-s growth time. We inferred that the large grains are preferred to the nanocrystalline form for nucleation on the Cu surface within the moderate-temperature zone in our PECVD process. When the graphene was transferred onto SiO_2_/Si, a large number of nanograins smaller than 100 nm were found on the substrate, as shown in Fig. 5g. When the growth time was extended to 30 s, the size of the large grains grew to 510 nm, and the nanograins were ~ 100 nm in size, as shown in Fig. 5c and h. At 40 s, the large grains of graphene further expanded to ~ 1.2 μm, and the nanograins were ~ 150 nm, as shown in Fig. 5d and i. This result illustrates that both large grains and nanocrystalline graphene grew simultaneously. At a growth time of 60 s, large graphene grains with an average size of 1.5 μm and nanocrystalline graphene grains 260 nm in size were obtained (Fig. 5c and j). At 90 s, the large grains were approximately 2 μm, and the nanograins increased in size to ~ 350 nm. At this time, the Cu foil was mostly fully covered with graphene, as shown in Fig 5f. The growth process described above shows the preferential nucleation of large graphene grains at moderate temperature (850 °C). A slight increase in the growth time resulted in the appearance of nanocrystalline graphene. The two sizes of grains subsequently grew simultaneously until the Cu foil was almost fully covered by graphene.

**Figure 6:**
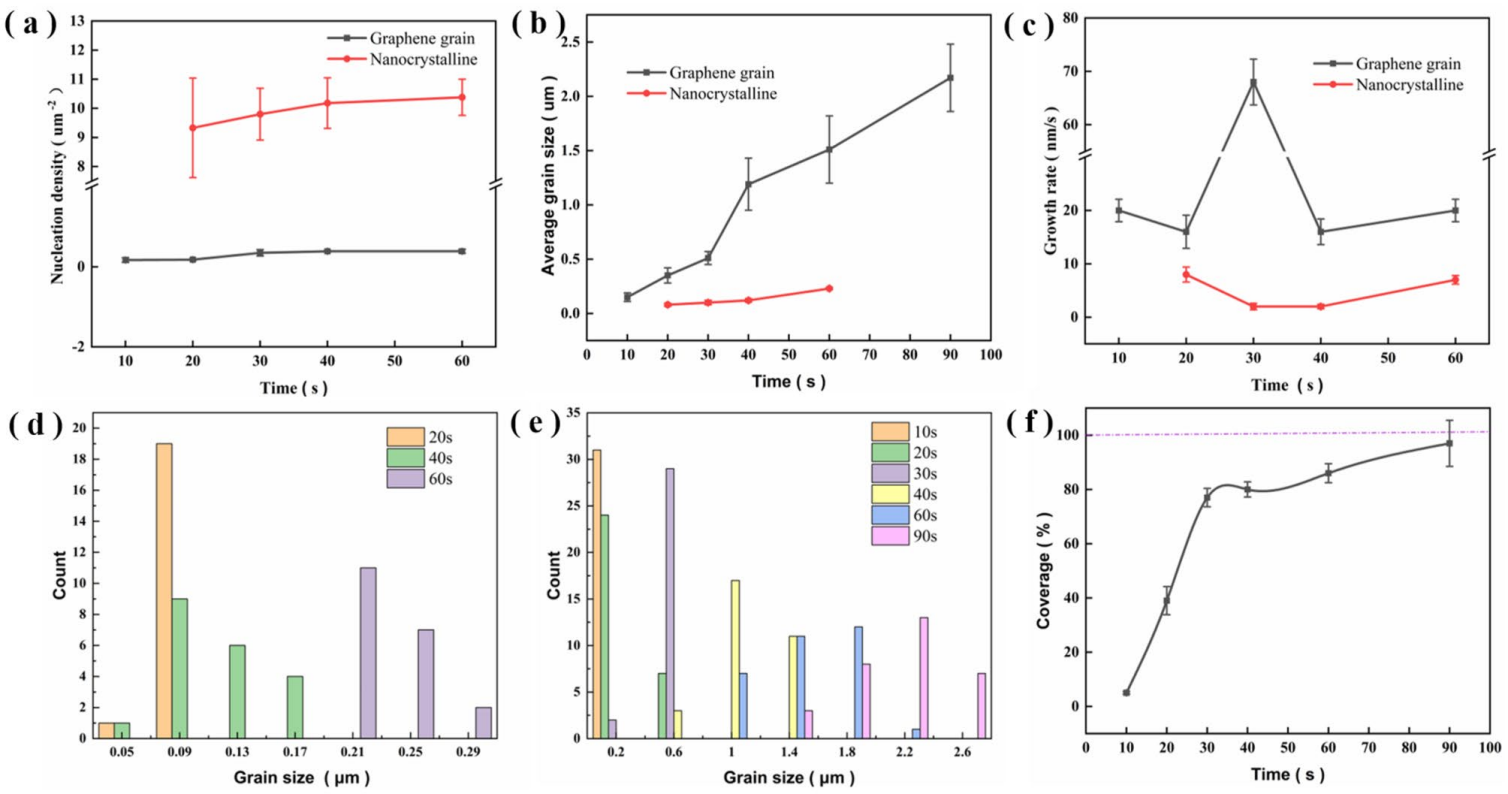
Time evolution of (**a**) the graphene nucleation density and (**b**) average grain size at 850 °C in PECVD. (**c**) Grain growth rate of graphene. The black line represents larger graphene grains, and the red line represents nanocrystalline graphene. Distribution of (**d**) nanocrystalline graphene and (**e**) graphene grains with the growth time. (**f**) Coverage of graphene grown on Cu with the growth time.

Figure 6 shows the nucleation density and average grain size of the two grain types at 850 °C with time. From Fig. 6a the large grains clearly nucleated before the nanograins and at a considerably lower nucleation density. As the growth time increased, the nucleation density of both grain types slightly increased, indicating that most of the nucleation of graphene in PECVD occurred during the early stage of growth. Figure 6b shows the change in the average grain size with time. As the growth proceeded, grains of both sizes continued to grow simultaneously until the copper foil was completely covered. The changes in the growth rate of the two types of grains are shown in Fig. 6c. The growth rate of large grains was higher than that of nanocrystalline graphene during the whole PECVD process. At 30 s, the large graphene grains grew rapidly, while the growth rate of nanocrystalline grains decreased, suggesting competitive growth between the two grain types from 20 to 40 s. The growth rate curves showed opposite trends between 20 and 40 s, suggesting competitive growth between the two grain types at this stage.

The growth process in the medium temperature region confirmed that the nucleation of large grains preceded the nucleation of small grains and grows at the same time. The nucleation and growth of large and small grains competed with each other for carbon atoms at a given carbon source flow rate. The plasma growth of graphene mainly includes four major processes: the dissociation of carbon sources, the transport of reactants to the substrate surface, the migration and nucleation growth of reactants on the substrate surface, and the desorption of the reaction byproducts on the substrate surface. Due to the greatly enhanced decomposition of the carbon source by the plasma, carbon supersaturation is very high, and the nucleation barrier is very low. The carbon clusters will spontaneously aggregate, and the growth barrier is determined by the deposition of carbon atoms and their diffusion rate on the metal surface. The surface roughness and chemical state of the copper foil greatly influence the deposition and diffusion of carbon atoms. Carbon atoms are more likely to coalesce and nucleate preferentially at high-energy sites such as the rough surface of the copper substrate, defect sites (grain boundaries, dislocations, steps, etc.) and impurities. And the follow-up of carbon atoms shows two kinds of behavior. The carbon atoms adsorbed near the nucleus could make the crystal grow, while the carbon atoms adsorbed at other locations were restricted by diffusion and migration, and tended to the termination of the growth of the crystal nucleus, eventually leading to the two completely different graphene grains. This was because that the energy at different locations on the surface of the copper substrate in the medium temperature region played a dominant role in the nucleation and growth of graphene rather than that of at lower or higher temperature.

The size distributions of the large grains and the nanograins are shown in Fig. 6d,e, respectively. The figures also illustrate the concurrent growth of the two grain types with time. The coverage of graphene grown at different times was statistically analyzed, as shown in Fig. 6f. During the rapid growth period (20–40 s), the graphene coverage increased sharply at first and then grew more slowly. The oval shape of the nanograins precluded the full-coverage growth of graphene.

### Growth model

**Figure 7:**
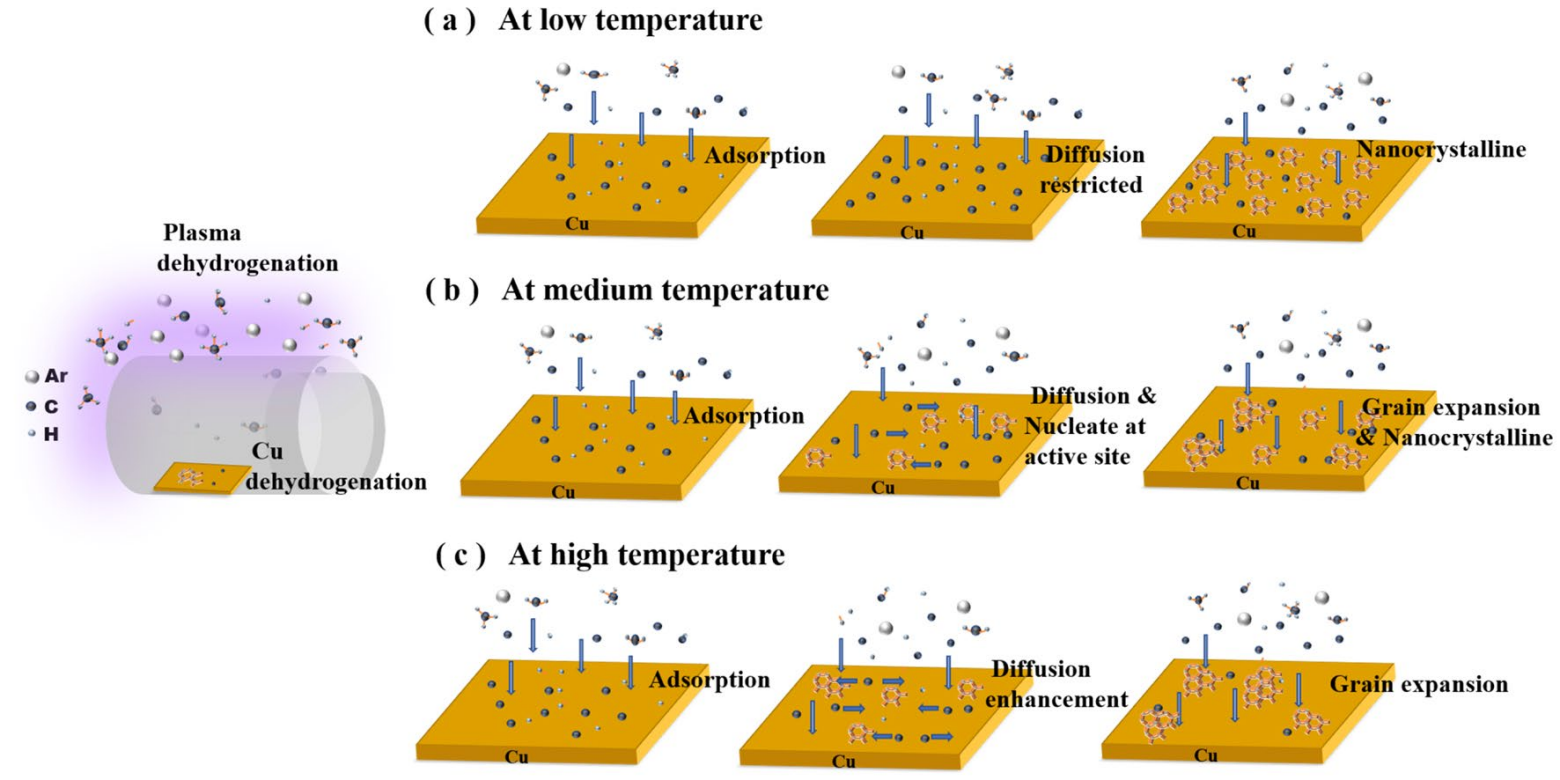
Schematic illustration of the nucleation and growth dynamics of PECVD at different temperatures.

The analysis above was used to develop a model to describe the nucleation and grain growth of graphene grown on a Cu substrate by PECVD at different temperatures. Figure 7a–c show the schematics of the models. The shielded placement method of Cu foils changed the transport mode of reactant gas to the surface of substrate and reduced the concentration of reactants on the substrate surface, which were beneficial for graphene growth. Second, temperature dependence of the nucleation density and grain growth can be divided into three regions. At low temperature, the surface catalysis of copper can be ignored, and the nucleation and growth of graphene mainly depend on the capture of carbon adatoms by supercritical nucleus, which was limited by the diffusion and migration of carbon species generated by plasma at lower temperatures.

Besides, the copper surface roughness and impurities are not well decreased, so it is easy to produce the growth of nanocrystalline graphene. At medium temperatures, the catalysis of copper substrate is improved to some extent, and the surface roughness of copper is reduced. So there will be carbon atoms preferentially nucleate at some high energy positions on the surface of the copper substrate, where the subsequent carbon atoms are easily attached, providing the raw material to fuel graphene growth. However, the carbon atoms adsorbed in other parts of the copper substrate nucleate and are difficult to grow up due to the influence of blocked diffusion and migration at low temperature. At this stage, the difference of energies on copper surface is an important and non-negligible factor that causes the difference of nucleation growth of graphene. At high temperatures, the catalysis of copper surface is stronger enough, the roughness of copper surface is reduced and the cleanliness is improved. According to the classic diffusion equation $D={D}_{0}{e}^{(-\frac{Q}{RT})}$, the diffusion of carbon species on the substrate surface was enhanced. In this stage, the influence of copper substrates is weakened (but still present), and the graphene nucleation is suppressed and the growth of grains are promoted.

## Conclusion

In summary, we surveyed the nucleation and grain growth of graphene grown on Cu during RF-PECVD. A reasonable shielding method for the placement of copper was employed and effectively reduced the damage to the graphene in the PECVD process, which could be beneficial for high-quality graphene growth. In addition, the graphene nucleation and grain size of graphene were significantly affected by the growth temperature. At low temperatures (670–760 °C), a mass of nanocrystalline grains was obtained. The nucleation density of nanocrystalline grains decreased and the grain size increased with increasing temperature at this stage. At moderate temperatures (790–850 °C), large grains and nanocrystalline graphene coexisted and grew simultaneously. In this case, as the growth time increased, the large graphene grains preferentially nucleated and grew rapidly, followed by the nucleation and growth of nanograins. At high temperatures (880 °C), there was no more nucleation and growth of nanocrystalline graphene, and grains with an average size of ~ 3 μm were generated. These results reveal the effects of temperature on nucleation and graphene growth during PECVD and provide guidance on the synthesis of high-quality graphene by PECVD.
